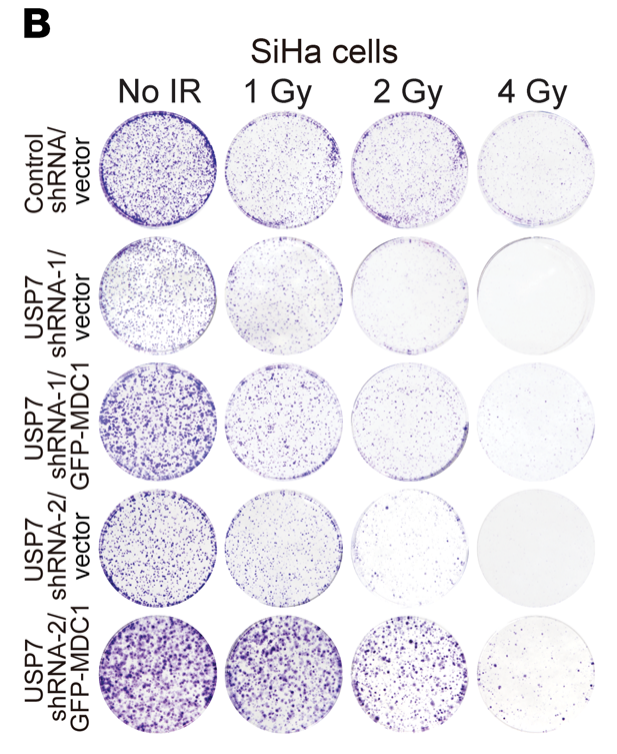# Corrigendum to Ubiquitin-specific protease 7 sustains DNA damage response and promotes cervical carcinogenesis

**DOI:** 10.1172/JCI197926

**Published:** 2025-09-02

**Authors:** Dongxue Su, Shuai Ma, Lin Shan, Yue Wang, Yuejiao Wang, Cheng Cao, Beibei Liu, Chao Yang, Liyong Wang, Shanshan Tian, Xiang Ding, Xinhua Liu, Na Yu, Nan Song, Ling Liu, Shangda Yang, Qi Zhang, Fuquan Yang, Kai Zhang, Lei Shi

Original citation: *J Clin Invest*. 2018;128(10):4280–4296. https://doi.org/10.1172/JCI120518

Citation for this corrigendum: *J Clin Invest*. 2025;135(17):e197926. https://doi.org/10.1172/JCI197926

In Figure 8B of the original article, there was an error in the colony formation plate for the 2 Gy-USP7 shRNA-2/vector sample, which was an inadvertent duplication of the 4 Gy-Control shRNA/vector sample. The corrected figure, based on the original source data, is provided below. The HTML and PDF versions of the paper have been updated.

The authors regret the error.

## Figures and Tables

**Figure:**